# Strengths use as a secret of happiness: Another dimension of visually impaired individuals' psychological state

**DOI:** 10.1371/journal.pone.0192323

**Published:** 2018-02-01

**Authors:** Shinichiro Matsuguma, Motoko Kawashima, Kazuno Negishi, Fumiya Sano, Masaru Mimura, Kazuo Tsubota

**Affiliations:** 1 Department of Ophthalmology, Keio University School of Medicine, Shinjuku-ku, Tokyo, Japan; 2 Clinical and Translational Research Center, Keio University Hospital, Shinjuku-ku, Tokyo, Japan; 3 Department of Neuropsychiatry, Keio University School of Medicine, Shinjuku-ku, Tokyo, Japan; Wuhan University, CHINA

## Abstract

It is well recognized that visual impairments (VI) worsen individuals’ mental condition. However, little is known about the positive aspects including subjective happiness, positive emotions, and strengths. Therefore, the purpose of this study was to investigate the positive aspects of persons with VI including their subjective happiness, positive emotions, and strengths use. Positive aspects of persons with VI were measured using the Subjective Happiness Scale (SHS), the Scale of Positive and Negative Experience-Balance (SPANE-B), and the Strengths Use Scale (SUS). A cross-sectional analysis was utilized to examine personal information in a Tokyo sample (N = 44). We used a simple regression analysis and found significant relationships between the SHS or SPANE-B and SUS; on the contrary, VI-related variables were not correlated with them. A multiple regression analysis confirmed that SUS was a significant factor associated with both the SHS and SPANE-B. Strengths use might be a possible protective factor from the negative effects of VI.

## Introduction

Visual impairments (VI) have been reported as a deteriorating factor in the field of ophthalmology. The majority of previous studies investigated the negative aspects of visual impairment, reporting VI as having a deleterious influence on individuals’ quality of life [1, 2], physical functioning [3, 4], self-esteem [5], socialization [6, 7], depression [8], and emotional distress [9]. Lam et al. even concluded that VI was associated with increased suicide risk [10]. Although these studies investigated both physical and psychological realities, the measures were limited only to negative aspects of VI and could not identify positive aspects of visually impaired persons’ lives.

In this context, positive psychology, a scientific study of human strengths, may play an important role in examining another side of persons with VI. In this field, strengths have been conceptualized in various ways over the last few decades [11–14]. While definitions differ, all have their roots in classic personality trait theory, regarding strengths as possessing a genetic and/or evolutionary dimension, which naturally makes individuals excel at certain types of performances [11]. To broadly cover these various descriptions of strengths and mark the point of agreement, Wood et al. recently proposed a new definition called “personal strengths,” defined as “the characteristics of a person that allow them to perform well or at their personal best” [15]. In addition, recent studies emphasize a clear distinction between using strengths and merely possessing them [15]. Strengths use is more highly associated with positive outcomes such as happiness and fewer depressive symptoms compared to mere strengths identification [16].

Considering these findings, we were intrigued to investigate the positive aspects of visual impaired individuals’ experiences, especially the associations between their personal strengths use, subjective happiness, and positive emotions in daily life.

We hypothesized that personal strengths use may be correlated with their subjective happiness and positive emotions, but we wondered if VI-related factors have more influence on their happiness and emotions than strengths use. Therefore, in an exploratory manner, we investigated the association between personal strengths use, subjective happiness, positive emotions, and VI-related factors to shed light on potential unidentified relationships.

## Methods

### Design, setting, and participants

The participants were recruited from the Japanese population of individuals who were legally classified as VI within the Japanese Physically Disabled Persons Welfare Act, and who attended the Japan Vocational Development Center for the Blind (JVDCB) between June and September 2016. Participants were excluded if they were aged less than 20 years. The Institutional Review Board of Keio University School of Medicine approved the research protocol, which followed the tenets of the Declaration of Helsinki. Informed consent was obtained from all participants by reading the informed consent form aloud and guiding them to sign the form.

### Outcome measures

#### Strengths Use Scale (SUS)

Strengths use was measured using the validated Japanese version of the SUS [17, 18]. The 14-item scale measures strengths use using a five-point Likert scale (ranging from 1–5, total range 14–70, see S1 Appendix), with higher values corresponding to greater use of strengths. The Japanese scale demonstrates good internal consistency (α = .94) and satisfactory test–retest reliability (r = .67) over four weeks [17].

#### Subjective Happiness Scale (SHS)

The SHS is a validated measurement that assesses subjective global happiness [19, 20]. The scale is a four-item measure of subjective happiness and rated on a seven-point Likert scale (ranging from 1–7), with higher values corresponding to higher subjective happiness (see S2 Appendix). Both internal consistency (α = .80 for males and α = .84 for females) and test–retest reliability (r = .86) over five weeks are scientifically sound [19].

#### Scale of Positive and Negative Experience (SPANE)

The SPANE was used to assess participants’ subjective feeling of well-being and ill-being [21, 22] by asking people to report their broad range of pleasant and unpleasant feelings. The SPANE consists of 12 items: six items assess positive feelings (SPANE-P) with a five-point Likert design (ranging from 1–5, total range 6–30) and the other six assess negative feelings (SPANE-N) (ranging from 1–5, total range 6–30). The balance of positive and negative feelings (SPANE-B) is calculated by subtracting SPANE-N from SPANE-P (total range 25–25) (see S3 Appendix). The subscales of the Japanese version demonstrate good internal consistency (α = .88–.91) and acceptable convergent validity by correlations with scores for life satisfaction, subjective happiness, optimism, pessimism, positive and negative affect, depression, anxiety, and psychological stress [23].

All the participants answered these questionnaires by responding to questions that a research member read aloud.

#### Personal information

Personal information was examined including sex, age, better eye-corrected visual acuity (BCVA), worse eye-corrected visual acuity (WCVA), history of VI, years of VI, legal Japanese visual impairment grades, employment status, education, annual income, systemic diseases, independent living, and support for daily activities. Both BCVA and WCVA were represented with a Logarithm of the Minimum Angle of Resolution (LogMAR) chart. A higher value on the LogMAR chart indicates poorer vision. The legal Japanese visual impairment grades were established and classified by the Japanese Physically Disabled Persons Welfare Act. There are six grades for vision-disabled certification based on visual function and the sum of the corrected visual acuity of both eyes [24]. A lower grade indicates a higher level of visual severity. Patients certified as visually disabled can receive publicly funded social services, financial support, and tax deductions according to their certification grade [24].

## Statistical analysis

The data obtained from participants’ responses for all questionnaires were used for statistical analysis. For the statistical analysis of their corrected visual acuity, counting fingers was categorized as an acuity of .004, hand motion as .002, light perception as .001, and no light perception as .0005. A simple regression analysis was used to investigate the association between the SHS or SPANE-B and other variables. Then, a multiple regression analysis was used to confirm the independent predictors of the SHS and SPANE-B, respectively; *p* < .05 was the threshold of significance for all analyses. All statistical analyses were performed using SPSS version 23 for Windows (SPSS Inc., Chicago, IL, USA).

## Results

### Participants’ sociodemographic, clinical, and subjective characteristics

Forty-four participants with VI were evaluated. Their mean age was 44.14 ± 12.80 years (range = 21–68 years). Participants mean BCVA and WCVA were 2.12 ± .99 (range: .10–3.30) and 2.55 ± 0.88 (0–3.30), respectively. Other characteristics are summarized in Table 1.

**Table 1:** Participants’ sociodemographic, clinical, and subjective characteristics (N = 44).

**Variables**	**N**	**%**
Sex		
Female	17	38.6
Male	27	61.4
History of visual impairments		
Congenital	14	31.8
Acquired	30	68.2
Disability grade*		
Grade 1	22	50.0
Grade 2	17	38.6
Grade 3	1	2.3
Grade 4	1	2.3
Grade 5	3	6.8
Employment status		
Employed	14	31.8
Unemployed	23	52.3
Temporary leave	7	15.9
Education		
≤ High school	13	29.5
> High school	31	70.5
Annual income** (yen/year)		
< 2 million	30	68.2
≥ 2 million, < 4 million	7	15.9
≥ 4 million, < 6 million	3	6.8
≥ 6 million	2	4.5
Systemic diseases		
Yes***	9	20.5
No	36	97.7
Live independently		
Yes	11	25.0
No	33	75.0
Support for daily activities		
Yes	26	59.1
No	18	40.9
**Variables**	**Mean**	SD**** **(Range)**
Age	44.14	12.80 (21–68)
Better eye-corrected visual acuity (LogMAR)	2.12	.99 (.10–3.30)
Worse eye-corrected visual acuity (LogMAR)	2.55	.88 (0–3.30)
Years of visual impairment	19.32	13.09 (1–49)

*Disability grades: The legal Japanese visual impairment grades. A lower grade indicates a higher level of visual severity.

**Missing values (n = 2).

***Depression (n = 1); chronic pancreatitis (n = 1), diabetes (n = 3), high blood pressure (n = 1), and chronic pain (n = 3).

****SD: standard deviation.

### Visually impaired persons’ SUS, SHS, and SPANE results

Participants strengths use (SUS), subjective happiness (SHS), and the balance between positive and negative feelings (SPANE-B) were 48.57 ± 10.53 (28–68), 4.54 ± 1.18 (1.25–7.00), and 2.98 ± 9.51 (-16–24), respectively (see in Table 2).

**Table 2:** Visually impaired persons’ SUS, SHS, and SPANE results.

Variables	Mean	SD	Range
Strengths Use Scale	48.57	10.53	28–68
Subjective Happiness Scale	4.54	1.18	1.25–7.00
SPANE-P	19.89	5.13	8–30
SPANE-N	16.91	5.72	6–28
SPANE-B	2.98	9.51	-16–24

SUS: Strength Use Scale; SHS: Subjective Happiness Scale; SPANE: Scale of Positive and Negative Experience; SPANE-P: SPANE-positive affects; SPANE-N: SPANE-negative affects; SPANE-B: Scale of Positive and Negative Experience-Positive—Scale of Positive and Negative Experience-Negative; SD: standard deviation.

### Association between subjective happiness and other variables

Subjective happiness or subjective well-being consists of two components: cognitive evaluation of one’s happiness, which was measured by the SHS, and the balance of positive feelings and negative feelings, which was measured by the SPANE-B. Therefore, we dealt with the SHS and SPANE-B separately. Using a simple regression analysis, we investigated the association between the SHS or SPANE-B and other variables. Significant positive associations were found between mean SHS score and mean SUS and education, whereas no correlation was found between the SHS and visual factors such as BCVA, WCVA, visual impairment grade, history of VI, or years of VI. More details can be seen in Table 3. Regarding SPANE-B, a positive association was only found with SUS; no relationship was found with visual factors (see Table 3).

**Table 3:** Simple regression analysis of the Subjective Happiness Scale (SHS) and Scale of Positive and Negative Experience-Balance (SPANE-B) with variables.

Dependent variable	SHS		SPANE-B	
Independent variables	β	P	β	P
Strengths Use Scale	.07*	< .001	.49*	< .001
Sex	.23	.53	4.18	.16
Age	.02	.09	.15	.18
BCVA	.25	.17	2.41	.10
WCVA	.36	.08	2.51	.13
Disability grade	-.05	.76	-.86	.52
History of VI	-.40	.30	-2.06	.51
Years of VI	.01	.36	.03	.78
Employment status	.77	.16	1.21	.78
Education	-.63	.11	.69	.83
Annual income	.16	.46	-.12	.94
Systemic diseases**	-.23	.59	1.60	.64
Independent living	.01	.99	.64	.85
Support for daily activities	.28	.45	3.82	.19

BCVA: better eye-corrected visual acuity; WCVA: worse eye-corrected visual acuity; VI: visual impairments.

*P-value < .05

**Depression (n = 1); chronic pancreatitis (n = 1), diabetes (n = 3), high blood pressure (n = 1), and chronic pain (n = 3).

To confirm the independent predictors of both the SHS and SPANE-B, we conducted a multiple regression analysis. Because of the small number of participants, we chose three variables (i.e., SUS, WCVA, and history of VI). The analysis demonstrated that the SUS was a crucial factor associated with both the SHS and SPANE-B (see Table 4).

**Table 4:** Multiple regression analysis of the Subjective Happiness Scale (SHS) and Scale of Positive and Negative Experience-Balance (SPANE-B).

Dependent variables	SHS		SPANE-B	
Independent variables	β	P	β	P
Strengths Use Scale	.07*	< .001	.46*	< .001
WCVA	.26	.11	1.77	.25
History of VI**	-.50	.10	-2.75	.32

WCVA: worse eye-corrected visual acuity

*P-value < .05

**Congenital or acquired.

**Fig 1:**
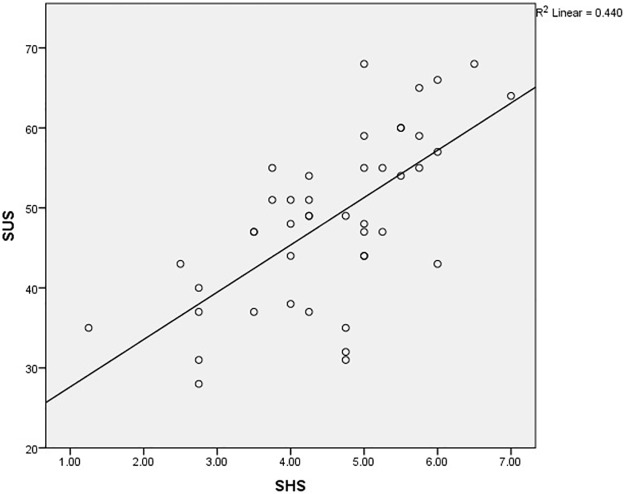
The correlation between the Strengths Use Scale (SUS) and Subjective Happiness Scale (SHS). SHS: Subjective Happiness Scale; SUS: Strengths Use Scale. SUS was strongly correlated to SHS (β = .07, p < .001).

**Fig 2:**
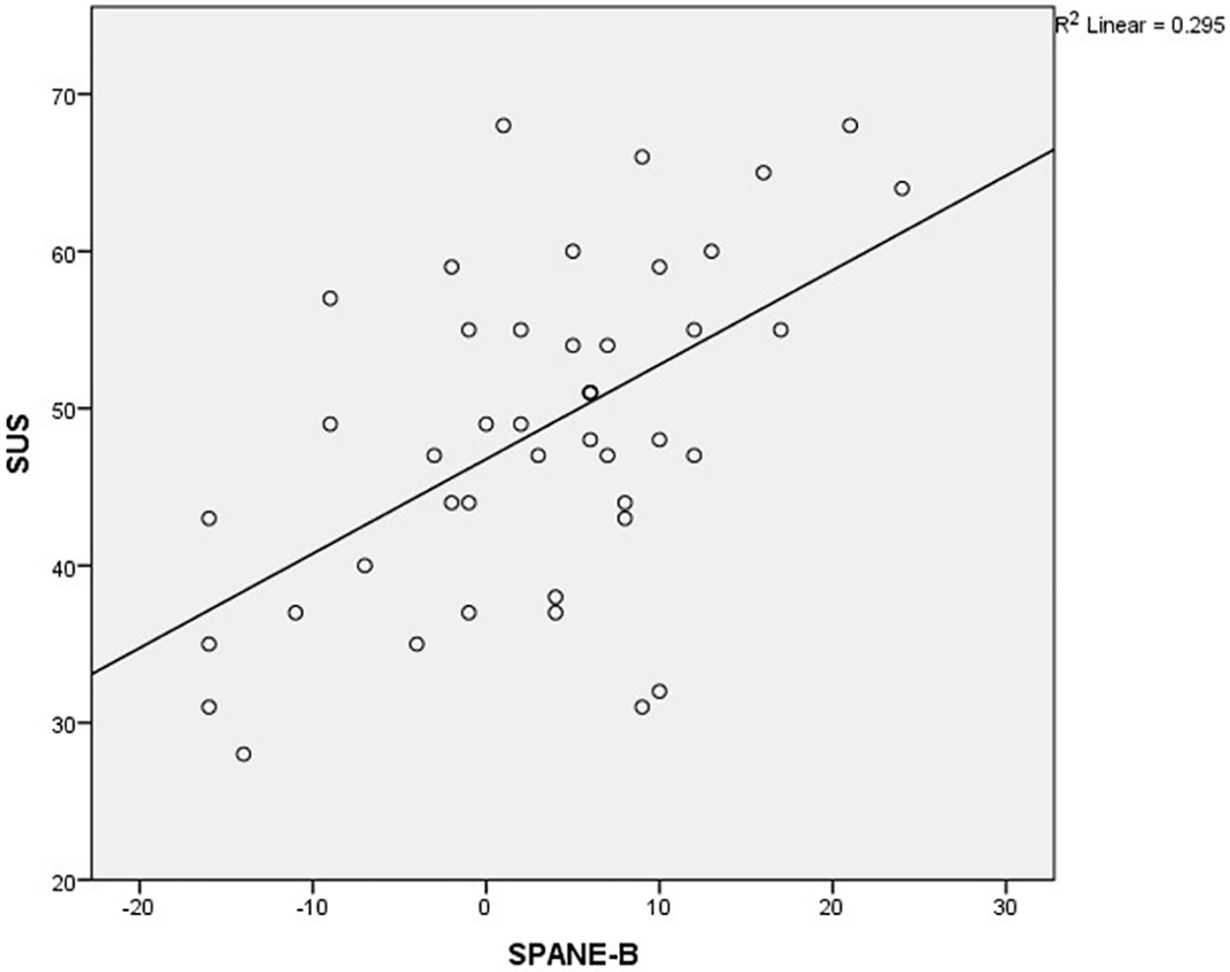
The correlation between the Strengths Use Scale (SUS) and Scale of Positive and Negative Experience (SPANE-B). SPANE-B: Scale of Positive and Negative Experience-Positive—Scale of Positive and Negative Experience-Negative; SUS: Strengths Use Scale. SUS was strongly correlated to SPANE-B (β = .46, p < .001).

The correlations between the SUS and SHS and the SUS and SPANE-B are shown in Figs 1 and 2 respectively.

## Discussion

This study was the first to investigate the association between personal strengths use and happiness among persons with VI. Surprisingly, this study revealed that strengths use was significantly associated with subjective happiness (β = .07, p < .001) and positive emotions (β = .46, p < .001) regardless of their visual condition (see Tables 3 and 4). This result was striking because previous literature emphasized a correlation between VI and negative psychological aspects [8–10]. To understand this contradictory finding, we suggest several possible explanations underlying this result.

First, irrespective of VI, using strengths itself may generate happiness and positive emotions. The association between strengths use and happiness or positive emotions within healthy participants has been reported in several prior studies [16, 25–27]. Peterson and Seligman suggested that strengths use leads to energizing experiences and elevated, sustainable well-being [12] and Layous and Lyubomirsky claimed that engaging in positive activities (i.e., using strengths) might increase the response to reward-relevant stimuli in the brain [28]. Besides, since strengths in positive psychology were originally based on the personality traits theory (i.e., positive traits), using strengths has nothing to do with external physical restrictions. Therefore, the present study indicates a significant association between strengths use and subjective happiness or positive emotions irrespective of one’s visual condition.

Additionally, strengths use may function as a protective factor against negative effects of VI. Human beings are naturally biased toward remembering the negative, because of selection for survival [29]. However, using strengths that participants already possess may produce “end-runs” around their perceived negativity [30] or focusing on the positive traits that remain in persons with disabilities leads to positive emotions that enhance resiliency [31]. Much prior positive psychology intervention research supports this buffering effect on negativity such as improving depressive symptom in patients with depression [30] or improving pain intensity as well as pain control in persons with chronic pain [32]. Considering these findings, strengths use might have played a protective role against the negative effects of VI.

**Fig 3:**
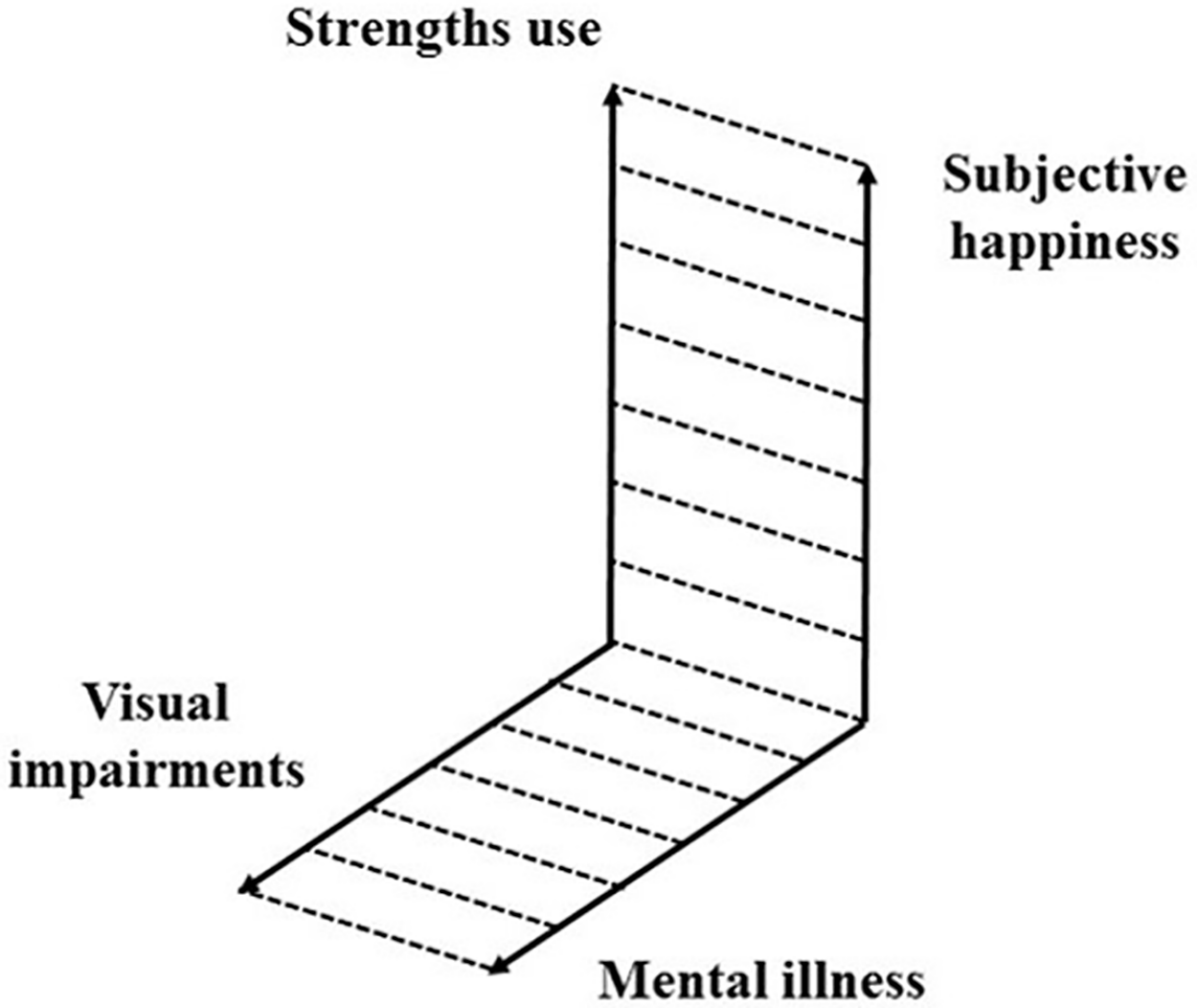
Two dimensions of the psychological states of persons with visual impairments. There are two dimensions of the internal state of persons with visual impairments. Previous literature focused on the horizontal dimension, which is related to negative experiences (the correlation between visual impairments and mental illness); however, this study revealed that there is another vertical dimension, which is related to positive experiences (the correlation between subjective happiness and strengths use).

Another plausible reason for this contradiction might be explained by the notion of a “mental health continuum” [33, 34]. Per this theory, mental health and mental illness are on different dimensions; having no mental illness does not mean one is necessarily happy. Huppert and Whittington also indicated that having good mental health is not the same as being without poor mental health [35]. Based on this theory, we could suggest that VI might be related to mental illness; however, VI can also become irrelevant to one’s sense of happiness if one’s strengths are used. To put it simply, VI and mental illness can be found on the same dimension; however, there is another dimension of internal states, where a sense of happiness coexists with strengths use on a different dimension (see Fig 3).

Regarding the external variables, social support is referred to as a predictor of happiness among persons with VI [36]. Since all participants in the present study received social support at the JVDCB, we could not identify the association between their subjective happiness and social support; however, participants’ mean SHS score (4.54 ± 1.18) was within the range of healthy samples in previous literature (range: 4.02 ± 0.93–5.62 ± 0.96) [19, 20], which implies that social support might be associated with subjective happiness in this study. Yet, even within the group of those who could access social support, the association between strengths use and subjective happiness was significant, which is a highlight of this study.

Aside from strengths use, the small range of visual severity (i.e., most participants’ visual severity was severe: 88.6% were Grade 1 and 2; see more details in Table 1) might have also caused this result.

It is worth noting that subjective happiness and positive emotions were not correlated with either employment status (β = .77, p = .16) or annual income (β = .77, p = .16), which contradicted a previous study that examined healthy samples [37]. This might be because the patients certified as visually disabled could receive financial support from the government. However, another reason may be due to the small number of participants or the very specific group, where 52.3% of participants were unemployed; therefore, this finding requires special attention and should be explored in future research.

## Limitations

Approximately 1,640,000 people suffer with VI in Japan [38] and 310,000 are officially registered as having VI as defined by the Physically Disabled Persons Welfare Act [39]. Therefore, our sample size was small, and all participants utilized a vocational institution, which had a full-time psychiatric social worker; consequently, baseline mental status might have been better compared to those who did not utilize such institutions.

In addition, since we used a cross-sectional design, we cannot infer a causal relationship between strengths use and subjective happiness or positive emotions. Further research should include an intervention study to provide confirmatory evidence for the association between strengths use and subjective happiness to clarify causality.

Furthermore, differences in their use of strengths and subjective happiness between pre- and post-blindness should be investigated in a future longitudinal study.

Additionally, we did not ask what strengths participants possessed; therefore, we could not identify what strengths might be specifically helpful to increase happiness. Further research should include the specific classification of strengths.

## Conclusions

In summary, although many studies have evaluated the relationship between VI and negative mental health aspects, this study sheds light on the positive aspects. We showed an association between strengths use, subjective happiness, and positive emotions. VI may cause emotional distress; however, this does not necessarily mean persons with VI are unhappy, especially when shielded by personal strengths.

## Supporting information

S1 Appendix Strengths Use Scale. (DOCX)

S2 Appendix Subjective Happiness Scale. (DOCX)

S3 Appendix Scale of Positive and Negative Experience. (DOCX)

S1 Dataset The raw data of the subjects. (XLSX)

## References

[1] Mangione CM, Gutierrez PR, Lowe G, Orav EJ, Seddon JM. Influence of age-related maculopathy on visual functioning and health-related quality of life. *Am J Ophthalmol* 1999; 128(1): 45–53. 10482093 10.1016/s0002-9394(99)00169-5

[2] Broman AT, Munoz B, Rodriguez J, Sanchez R, Quigley HA, Klein R, et al The impact of visual impairment and eye disease on vision-related quality of life in a Mexican-American population: proyecto VER. *Invest Ophthalmol Vis Sci* 2002; 43(11): 3393–3398. 12407148

[3] Swanson MW, McGwin G. Visual impairment and functional status from the 1995 National Health Interview Survey on Disability. *Ophthalmic Epidemiol* 2004; 11(3): 227–239. doi: 10.1080/09286580490514540 15370554 10.1080/09286580490514540

[4] Steinberg EP, Tielsch JM, Schein OD, Javitt JC, Sharkey P, Cassard SD, et al The VF-14. An index of functional impairment in patients with cataract. *Arch Ophthalmol* 1994; 112(5): 630–638. 8185520 10.1001/archopht.1994.01090170074026

[5] Papadopoulos K, Montgomery AJ, Chronopoulou E. The impact of visual impairments in self-esteem and locus of control. *Res Dev Disabil* 2013; 34(12): 4565–4570. doi: 10.1016/j.ridd.2013.09.036 24176255 10.1016/j.ridd.2013.09.036

[6] Scott IU, Smiddy WE, Schiffman J, Feuer WJ, Pappas CJ. Quality of life of low-vision patients and the impact of low-vision services. *Am J Ophthalmol* 1999; 128(1): 54–62. 10482094 10.1016/s0002-9394(99)00108-7

[7] Wang JJ, Mitchell P, Smith W, Cumming RG, Attebo K. Impact of visual impairment on use of community support services by elderly persons: the Blue Mountains Eye Study. *Invest Ophthalmol Vis Sci* 1999; 40(1): 12–19. 9888421

[8] Ribeiro MV, Hasten-Reiter HN Junior, Ribeiro EA, Juca MJ, Barbosa FT, Sousa-Rodrigues CF. Association between visual impairment and depression in the elderly: a systematic review. *Arq Bras Oftalmol* 2015; 78(3): 197–201. doi: 10.5935/0004-2749.20150051 26222114 10.5935/0004-2749.20150051

[9] Cho GE, Lim DH, Baek M, Lee H, Kim SJ, Kang SW. Visual impairment of Korean population: Prevalence and impact on mental health. *Invest Ophthalmol Vis Sci* 2015; 56(8): 4375–4381. 26176874 10.1167/iovs.15-16462

[10] Lam BL, Christ SL, Lee DJ, Zheng DD, Arheart KL. Reported visual impairment and risk of suicide: the 1986–1996 national health interview surveys. *Arch Ophthalmol* 2008; 126(7): 975–980. doi: 10.1001/archopht.126.7.975 18625946 10.1001/archopht.126.7.975 PMC2630284

[11] Biswas-Diener R, Kashdan TB, Minhas G. A dynamic approach to psychological strength development and intervention. *J Posit Psychol* 2011; 6(2): 106–118.

[12] Peterson C, Seligman MEP. Character strengths and virtues: A handbook and classification. New York: Oxford University Press; 2004.

[13] Hodges T, Clifton D. Strenguhs-based development in practice In: Linley PA, Joseph S, editors: Positive practice in psychology. Hoboken: Wiley; 2004 pp. 256–268.

[14] Linley PA, Harrington S. Playing to your strengths. *The psychologist* 2006; 19: 85–89.

[15] Wood AM, Linley PA, Maltby J, Kashdan TB, Hurling R. Using personal and psychological strengths leads to increases in well-being over time: A longitudinal study and the development of the strengths use questionnaire. *Pers Individ Dif* 2011; 50(1): 15–19.

[16] Seligman ME, Steen TA, Park N, Peterson C. Positive psychology progress: empirical validation of interventions. *Am Psychol* 2005; 60(5): 410–421. doi: 10.1037/0003-066X.60.5.410 16045394 10.1037/0003-066X.60.5.410

[17] Takahashi M, Morimoto Y. [Development of Japanese version of the Strength Use Scale (SUS).] *Jpn J Res Emot* 2015; 22(2): 94–99. Japanese.

[18] Govindji R, Linley P. Strengths use, self-concordance and well-being: Implications for strengths coaching and coaching psychologists. *International Coaching Psychology Review* 2007; 2: 143–153.

[19] Shimai S, Otake K, Utsuki N, Ikemi A, Lyubomirsky S. [Development of a Japanese version of the Subjective Happiness Scale (SHS), and examination of its validity and reliability.] *Nihon Koshu Eisei Zasshi* 2004; 51(10): 845–853. Japanese. 15565993

[20] Lyubomirsky S, Lepper HS. A measure of subjective happiness: Preliminary reliability and construct validation. *Soc Indic Res* 1999; 46(2): 137–155.

[21] Diener E, Wirtz D, Tov W, Kim-Prieto C, Choi D-w, Oishi S, et al New well-being measures: Short scales to assess flourishing and positive and negative feelings. *Soc Indic Res* 2010; 97(2): 143–56.

[22] Li F, Bai X, Wang Y. The Scale of Positive and Negative Experience (SPANE): Psychometric properties and normative data in a large Chinese sample. *PLoS One* 2013; 8(4): e61137 doi: 10.1371/journal.pone.0061137 23573297 10.1371/journal.pone.0061137 PMC3616102

[23] Sumi K. Reliability and validity of Japanese versions of the Flourishing Scale and the Scale of Positive and Negative Experience. *Soc Indic Res* 2014; 118(2): 601–615.

[24] Kawashima M, Hiratsuka Y, Nakano T, Tamura H, Ono K, Murakami A, et al The association between legal Japanese visual impairment grades and vision-related quality of life. *Japanese journal of ophthalmology* 2016; 60(3): 219–225. doi: 10.1007/s10384-016-0437-1 26972696 10.1007/s10384-016-0437-1

[25] Ghielen STS, van Woerkom M, Christina MM. Promoting positive outcomes through strengths interventions: A literature review. *J Posit Psychol* 2017; 1–13. Available from: http://www.tandfonline.com/doi/abs/10.1080/17439760.2017.1365164?journalCode=rpos20

[26] Gander F, Proyer RT, Ruch W, Wyss T. Strength-based positive interventions: Further evidence for their potential in enhancing well-being and alleviating depression. *J Happiness Stud* 2013; 14(4): 1241–1259.

[27] Douglass R, Duffy R. Strengths use and life satisfaction: A moderated mediation approach. *J Happiness Stud* 2015; 16(3): 619–632.

[28] Layous K, Lyubomirsky S. The how, why, what, when, and who of happiness: Mechanisms underlying the success of positive interventions In: Gruber J, Moscowitz JT, editors: Positive emotion: Integrating the light sides and dark sides. New York: Oxford University Press; 2014 pp. 473–495.

[29] Baumeister RF, Bratslavsky E, Finkenauer C, Vohs KD. Bad is stronger than good. *Rev Gen Psychol* 2001; 5(4), 323–370.

[30] Seligman ME, Rasid T, Parks AC. Positive psychotherapy. Am Psychol 2006; 61(8): 774–788. doi: 10.1037/0003-066X.61.8.774 17115810 10.1037/0003-066X.61.8.774

[31] Dana SD, Gitendra U, Timothy RE, Alissa L, Brittany B. A positive psychology of physical disability: Principles and progress In: Wehmeyer ML, editor. The oxford handbook of positive psychology and disability. New York: Oxford University Press; 2013: pp. 427–441.

[32] Peters ML, Smeets E, Feijge M, van Breukelen G, Andersson G, Buhrman M, et al Happy despite pain: A randomized controlled trial of an 8-week internet-delivered positive psychology intervention for enhancing well-being in patients with chronic pain. *Clin J Pain* 2017; 33(11): 962–975 doi: 10.1097/AJP.0000000000000494 28379873 10.1097/AJP.0000000000000494 PMC5636048

[33] Keyes CL. The mental health continuum: from languishing to flourishing in life. *J Health Soc Behav* 2002; 43(2): 207–222. 12096700

[34] Keyes CL. Mental illness and/or mental health? Investigating axioms of the complete state model of health. *J Consult Clin Psychol* 2005; 73(3): 539–548. doi: 10.1037/0022-006X.73.3.539 15982151 10.1037/0022-006X.73.3.539

[35] Huppert FA, Whittington JE. Evidence for the independence of positive and negative well-being: implications for quality of life assessment. *Br J Health Psychol* 2003; 8(Pt 1): 107–122. doi: 10.1348/135910703762879246 12643820 10.1348/135910703762879246

[36] Papadopoulos K, Papakonstantinou D, Koutsoklenis A, Koustriava E, Kouderi V. Social support, social networks, and happiness of individuals with visual impairments. *Rehabil Couns Bull* 2015; 58(4): 240–249.

[37] Kahneman D, Deaton A. High income improves evaluation of life but not emotional well-being. *Proc Natl Acad Sci U S A* 2010; 107(38): 16489–16493. doi: 10.1073/pnas.1011492107 20823223 10.1073/pnas.1011492107 PMC2944762

[38] Yamada M, Hiratsuka Y, Roberts CB, Pezzullo ML, Yates K, Takano S, et al Prevalence of visual impairment in the adult Japanese population by cause and severity and future projections. *Ophthalmic Epidemiol* 2010; 17(1): 50–57. doi: 10.3109/09286580903450346 20100100 10.3109/09286580903450346

[39] Nakae K, Masuda K, Senoo T, Sawa M, Kanai A, Ishibashi T. Aging society and eye disease: a recent epidemiological study on underlying diseases responsible for visual impairment. *Geriatr Med* 2006; 44: 1221–1224.

