# Supplementary material for: Strengths use as a secret of happiness: Another dimension of visually impaired individuals' psychological state
Source: PLoS One. 2018 Feb 1;13(2):e0192323. doi: 10.1371/journal.pone.0192323 (PMC5794170; doi:10.1371/journal.pone.0192323)
Supplement: S1 Appendix — (DOCX) [file pone.0192323.s001.docx]

**S1 Appendix. Strengths Use Scale**

| 1 | I am regularly able to do what I do best. |
| --- | --- |
| 2 | I always play to my strengths. |
| 3 | I always try to use my strengths. |
| 4 | I achieve what I want by using my strengths. |
| 5 | I use my strengths everyday. |
| 6 | I use my strengths to get what I want out of life. |
| 7 | My work gives me lots of opportunities to use my strengths. |
| 8 | My life presents me with lots of different ways to use my strengths. |
| 9 | Using my strengths comes naturally to me. |
| 10 | I find it easy to use my strengths in the things I do. |
| 11 | I can use my strengths in lots of different situations. |
| 12 | Most of my time is spent doing the things that I am good at doing. |
| 13 | Using my strengths is something I am familiar with. |
| 14 | I can use my strengths in lots of different ways. |

Note: Items for the Strengths Use Scale (Govindji & Linley, 2007) and loadings from our exploratory factor analysis. The scale was administered with the instructions, ‘‘The following questions ask you about your strengths, that is, the things that you are able to do well or do best.” Participants responded using a 7-point Likert scale ranging from 1 = *strongly disagree* to 7 = *strongly agree*.
