# Supplementary material for: Strengths use as a secret of happiness: Another dimension of visually impaired individuals' psychological state
Source: PLoS One. 2018 Feb 1;13(2):e0192323. doi: 10.1371/journal.pone.0192323 (PMC5794170; doi:10.1371/journal.pone.0192323)
Supplement: S2 Appendix — (DOCX) [file pone.0192323.s002.docx]

**S2 Appendix. Subjective Happiness Scale**

| 1 | In general, I consider myself  (not a very happy person) 1 2 3 4 5 6 7 (a very happy person) |
| --- | --- |
| 2 | Compared with most of my peers, I consider myself  (less happy) 1 2 3 4 5 6 7 (more happy) |
| 3 | Some people are generally very happy. They enjoy life regardless of what is going on, getting the most out of everything. To what extent does this characterization describe you?  (not at all) 1 2 3 4 5 6 7 (a great deal) |
| 4 | Some people are generally not very happy. Although they are not depressed, they never seem as happy as they might be. To what extent does this characterization describe you?  (not at all) 1 2 3 4 5 6 7 (a great deal) |

To score the scale, the 4th item was reverse coded (i.e., 7 = 1, 6 = 2, etc.) and the means were summed.
