# Supplementary material for: Strengths use as a secret of happiness: Another dimension of visually impaired individuals' psychological state
Source: PLoS One. 2018 Feb 1;13(2):e0192323. doi: 10.1371/journal.pone.0192323 (PMC5794170; doi:10.1371/journal.pone.0192323)
Supplement: S3 Appendix — (DOCX) [file pone.0192323.s003.docx]

**]S3 Appendix. Scale of Positive and Negative Experience**

Please think about what you have been doing and experiencing during the past four weeks; then, using the scale below, report how much you experienced each of the following feelings. For each item, select a number from 1 to 5, and indicate that number on your response sheet.

1. Very Rarely or Never

2. Rarely

3. Sometimes

4. Often

5. Very Often or Always

Positive

Negative

Good

Bad

Pleasant

Unpleasant

Happy

Sad

Afraid

Joyful

Angry

Contented

© Copyright by Ed Diener and Robert Biswas-Diener, January 2009.
